# Sympathetic skin response in patients with systemic sclerosis and rheumatoid arthritis

**DOI:** 10.1186/s41983-018-0044-9

**Published:** 2018-11-27

**Authors:** Reda Badry, Rania M. Gamal, Manal M. Hassanien, Mohamed Abd El Hamed, Nevin Hammam, Bastawy M. El Fawal

**Affiliations:** 1 Department of Neurology and Psychiatry, Assiut University Hospital, Post Box: 711526, Assiut, Egypt; 2 Rheumatology and Rehabilitation Department, Assiut University Hospital, Assiut, Egypt; 3 Department of Neurology and Psychiatry, Aswan University Hospital, Aswan, Egypt

**Keywords:** Sympathetic skin response (SSR), Systemic sclerosis (SSc), Rheumatoid arthritis (RA), Autonomic dysfunction (AD)

## Abstract

**Background:**

Sympathetic skin response (SSR) is a technique to assess the sympathetic cholinergic pathways. Sympathetic dysfunction may participate in the development of pain, which is the major complaint in patients with systemic sclerosis (SSc) and rheumatoid arthritis (RA).

**Objectives:**

In this study, we aimed to assess the autonomic dysfunction in patients with (SSc) and (RA) using SSR as a simple neurophysiologic test.

**Methods:**

Palmar SSR to median nerve electrical stimulation was recorded in 21 patients with SSc, 39 patients with RA, and in 60 healthy age and sex-matched control subjects.

**Results:**

Palmar SSR to median nerve stimulation (of SSc patients and RA patients) shows significantly delayed latency and reduced amplitude in comparison to the control group. SSR of SSc patients has significantly delayed latency and reduced amplitude when compared to RA patients. Moreover, six SSc patients have delayed SSR in spite of the absence of manifestations of polyneuropathy.

**Conclusions:**

Patients with SSc and RA have features of autonomic dysfunction with more affection of SSc patients.

## Introduction

Sympathetic skin response (SSR) is a potential generated by sweat glands in response to a variety of stimulation [1]. This technique records changes in skin conductance after activation of sweat glands in areas of the skin that are rich in eccrine glands (commonly palmar and plantar sites) under the neural control of sympathetic cholinergic (sudomotor) fibers [2, 3]. SSR potentials can be recorded in response to various stimuli, for example, electric peripheral nerve stimulation, acoustic stimulation, or magnetic stimulation of nerves or the brain [4]. SSR potential has a waveform that habituates with closely repeated stimuli [5]. It has been used to study the peripheral sympathetic fibers in diseases of peripheral nerves [6]. Neuropsychiatric manifestations (NPM), especially autonomic dysfunction, are frequent in patients with systemic sclerosis (SSc) and rheumatoid arthritis (RA); however, there are no consistent clinical criteria or specific tests to assess them [7]. Neuropathic features such as trigeminal neuralgia, peripheral neuropathy, and subacute combined degeneration of the spinal cord (as a consequence of vitamin B_12_ malabsorption) are well recognized in systemic sclerosis and rheumatoid arthritis [8]. Degenerative and regenerative changes of small nerve fibers are found in both involved and clinically uninvolved skin of patients with SSc [7]. Some manifestations of SSc, such as abnormal esophageal motility, gastrointestinal dysfunction, and microcirculatory impairment, are usually attributed to autonomic dysfunction (AD) [9]. Pathological changes involving the autonomic nerves may even precede clinical involvement of the skin by scleroderma [10]. It is possible that several phenomena may play overlapping roles in the development of the symptoms and signs of RA and SSc, and one of these may be dysfunction of the autonomic nervous system (ANS). Pain is the major symptom, and recent studies have considered this to be partially attributed to neuropathic pain related to central nervous system sensitization [11]. Studies have also shown the association of this pain with sympathetic neurotransmitters and increased function in the sympathetic nervous system of patients with many rheumatologic disorders [12]. Stress response plays an important role in the symptoms, and it is believed that the ANS has a close relationship with the efficiency of the stress response and the hypothalamic-pituitary-adrenal axis (HPA) [11]. Based on this, studying sympathetic function (using SSR) may add value in understanding some manifestations of RA and SSc in clinical practice.

In this study, we aimed to assess the autonomic dysfunction in patients with SSc and RA using SSR as a simple neurophysiologic test. We hypothesize that SSR potentials will be of delayed latency and reduced amplitude in patients with SSc and RA.

### Subjects and methods

#### Subjects

Twenty-one female patients with SSc and 39 female patients with RA were included in this study. Those patients were seen in the outpatient clinic of the Department of Rheumatology and Rehabilitation of Assiut University Hospital and Aswan University Hospital. The average age of the 21 female patients with SSc is 42.6 ± 5.7 years (range from 27 years to 60 years), while the average age of the 39 female patients with RA is 43.15 ± 8.2 years (range from 25 years to 51 years).

We excluded patients receiving medications that interfere with autonomic functions, for example, beta-blocker and amitriptyline, and also patients with severe skin lesions that interfere with the technical maneuver of the study.

Informed written consent (to participate in the study) was obtained from the patients. The ethical committee of the Faculty of Medicine, Assiut University Hospital, approved this study. All the involved patients fulfilled the 1982 revised criteria of the American College of Rheumatology (ACR) for the diagnosis of SSc and RA. Sixty age and sex-matched control subjects were involved in the study with an average age of 40.32 ± 5.9 years (range from 26 years to 53 years).

##### Some clinical data of the involved patients

Tables 1 and 2 show some clinical data of patients with SSc and patients with RA respectively.

**Table 1:** Clinical data of SSc patients

Symptoms and signs	Number of patients (%)
Skin tightness	21 (100%)
Calcinosis	2 (9.5%)
Telangectasia	6 (28.6%)
Dryness	1 (4.7%)
Raynaud’s phenomenon	19 (90.5%)
Digital ulcers	13 (61.9%)
Arthritis	11 (52.4%)
Gangrene	1 (4.7%)
Pitting scars	18 (85.7%)
Contractures	12 (57.1%)
Acrolysis	12 (57.1%)
Hand closure	18 (85.7%)
Mouth opening	19 (90.5%)
Weakness	3 (14.3%)
Myalgia	9 (42.9%)
Dyspnea	10 (47.6%)

*SSc* systemic sclerosis

**Table 2:** Clinical criteria of RA patients

Symptoms and signs	Number of patients (%)
Swollen joints	31 (79.5%)
Tender joints	33 (84.6%)
Other system involvement	24 (61.5%)
In remission	5 (12.8%)
High global score	12 (30.8%)
Moderate global score	19 (48.7%)
Low global score	8 (20.5%)

*RA* rheumatoid arthritis

## Methods

### Procedure of sympathetic skin response (SSR)

During the test, the subject sat in a comfortable armchair and was relaxed but not asleep. The room temperature was maintained at 24 ± 0.5 °C. Skin temperature of the examined limbs remained unchanged (33 ± 0.5 °C) all over the time of the test. This protocol was followed because the temperature is known to affect conduction velocity of unmyelinated sympathetic fibers [13]. The SSR technique and recordings were based on the procedures described by Shahani and his colleagues [14]. An investigator performed all the steps of the experiments, and care was taken to avoid all external stimulation.

### Electrical stimulation

The SSR was recorded from the palmar surface of both left and right hands for patients and control groups (240 hands in total). We used surface electrodes (Ag- Ag Cl) and Nihon Khoden machine (Neuropack X1, MEB 2300, 6 and 12 channels EMG/EP Measuring System, easy database management with Neuro-Work-bench software). The active electrode was placed on the palmar aspects of the hands, above the third metacarpal bones (at 3 cm from the distal end). The reference electrode was placed on the corresponding area of the dorsal aspect of the examined hand. The ground electrode was placed at the distal skin crease at the wrist area. All subjects were stimulated at the median nerve at the wrist ipsilateral to the recording electrode. Each stimulus consisted of a single electric pulse (width 0.5 ms). The stimulus intensity was set to be 1.5 times the motor threshold of the stimulated nerve, and it was delivered at the wrist at an irregular interval (30 to 35 s). The traces were recorded from 0.5 s before to 8 s after the trigger stimulus. The sweep speed was 1000 ms/D. The amplifiers’ sensitivity was 100 μV/div, and the amplifier filters were set at 0.5 Hz and 2 kHz for the low- and high-frequency filters respectively. Five trials were recorded from the palm with the largest amplitude, and shortest latency was taken. Peak-to-peak amplitude is measured, and latency was measured at the start point of either initial negativity or positivity of the baseline. To minimize noise from spontaneous potentials, the operator monitored the baseline on an oscilloscope before releasing the electric stimulus. Recording long latency responses (with low-frequency components) requires a very slow sweep (0.5~1 s per division), a high gain (100 μv per division), and a wide band pass (0.16~3 kHz).

#### Statistical analysis

Data were fed to the computer and analyzed using IBM SPSS software package version 20.0 [15]. Qualitative data were described using number and percent. Quantitative data were described using range (minimum and maximum), mean, and standard deviation (SD). The significance of the obtained results was judged at *P* value ≤ 0.05. The used tests were Student’s *t* test (for normally quantitative variables, to compare between two studied groups), *F* test (ANOVA) (for normally quantitative variables, to compare between more than two groups), and post hoc test (LSD) (for pairwise comparisons).

## Results

### Control group

All the normal 60 subjects had a palmar SSR to median nerve stimulation for both palms.

### Systemic sclerosis group

This group includes 14 patients with a limited form of SSc and 7 patients with a diffuse form of SSc. Disease duration of patients of this group is 13.45 ± 3.1 years. All patients had a palmar SSR to median nerve stimulation for both palms.

### Rheumatoid arthritis group

Disease duration of patients of this group is 14.31 ± 3.2 years. All patients had a palmar SSR to median nerve stimulation for both palms.

In total, we examined 240 writs (42 for Ssc, 78 for RA, and 120 for control). Average latency and amplitude of palmar SSR to median nerve stimulation for the three examined groups (control group, SSc group, and RA group) are given in Table 3. It is noted that there is significantly delayed latency and reduced amplitude of SSR potentials in patient groups (RA group and SSc group) when compared to the control group. Moreover, it is noted that SSR potentials of SSc patients show more delayed latency and smaller amplitude than that of patients with RA. Additionally, we found that the SSR potentials of the six patients with SSc show 2.5 SD more delay in latency and reduction in amplitude than that of the control group, in spite of the absence of polyneuropathy.

**Table 3:** Average values of SSR in the three groups (control group, SSc group, and RA group)

	Control group (I) (*N* = 60)	SSc group (II) (*N* = 21)	RA group (III) (*N* = 39)	Overall *P* value
Average latency of SSR (ms)	1545 ± 132	1890 ± 153	1722 ± 121	0.03*
Average amplitude of SSR (mv)	2.77 ± 1.4	1.21 ± 0.7	1.71 ± 0.11	0.04*
Post hoc test	(I) vs (II) 0.04*	(II) vs (III) 0.02*	(III) vs (I) 0.05*	

*SSR* sympathetic skin response, *SSc* systemic sclerosis, *RA* rheumatoid arthritis

*Statistically significant

## Discussion

The etiology of systemic sclerosis and rheumatoid arthritis remains unknown. Some manifestations like vasomotor instability, Raynaud’s phenomenon, abnormal esophageal motility, and bowel involvement may be attributed to autonomic dysfunction [13]. The precise nature of the autonomic dysfunction is unclear. It is recognized that sympathetic innervations may remain intact even in the presence of severe parasympathetic damage in RA and SSc [7]. In this study, the results suggest that there is an autonomic dysfunction in patients with SSc and RA in comparison to the control group regarding latency and amplitude of SSR potentials. With this finding, we can assume that autonomic dysfunction is one of the etiological factors linked to the development of microvascular manifestations of RA and SSc, and also, it may play a role in the development of pain symptoms of those diseases.

Interestingly, in this research, we found six patients with SSc having impaired SSR parameters (latency and amplitude) in spite of the absence of peripheral neuropathy. This suggests that the dysfunction of the autonomic nervous system may occur as an isolated neurological insult. This finding is consistent with Sonnex and his colleagues and Adlan and his colleagues [7, 8] who stated that autonomic dysfunction may take place even before the occurrence of polyneuropathy in patients with SSc in contrast to the autonomic failure associated with other connective tissue diseases, such as RA, in which, manifestations of demonstrable peripheral neuropathy usually precede.

Areas of generalized and localized scleroderma usually show histopathological lesions specifically of nerve fibers and ganglia [13, 16]. As, apparently, normal skin in scleroderma can show abnormal neurohistology, it is possible that these neuropathic lesions represent a primary pathogenic process [17].

Fries and his co-workers, who measured skin chronaxy, suggested that there is a primary injury of the autonomic nervous system at ganglionic and higher control levels [10]. Fries also demonstrated the increased skin resistance to the passage of weak electric current in the digits of patients with SSc [10]. This was interpreted as autonomic underactivity, and it was suggested that this might be a compensatory mechanism to increase blood flow to ischemic areas [18].

We found some obstacles during the performance of this study, like difficulty in recruitment of more patients and some technical difficulty in applying the stimulator to patients with skin lesions at the site of the electric stimulation.

## Conclusions

Patients with SSc and RA have features of autonomic dysfunction with more affection of SSc patients. This finding supports the notion that sympathetic dysfunction in those patients may play a role in the development of pain symptoms.

## Abbreviations

ACR: American College of Rheumatology
AD: Autonomic dysfunction
ANS: Autonomic nervous system
HPA: Hypothalamic-pituitary-adrenal axis
NPM: Neuropsychiatric manifestations
RA: Rheumatoid arthritis
SD: Standard deviation
SSc: Systemic sclerosis
SSR: Sympathetic skin response
